# Metabolomic, transcriptomic and genetic integrative analysis reveals important roles of adenosine diphosphate in haemostasis and platelet activation in non‐small‐cell lung cancer

**DOI:** 10.1002/1878-0261.12568

**Published:** 2019-09-30

**Authors:** Long T. Hoang, Clara Domingo‐Sabugo, Elizabeth S. Starren, Saffron A.G. Willis‐Owen, Deborah J. Morris‐Rosendahl, Andrew G. Nicholson, William O. C. M. Cookson, Miriam F. Moffatt

**Affiliations:** ^1^ National Heart and Lung Institute Faculty of Medicine Imperial College London UK; ^2^ Clinical Genetics and Genomics Royal Brompton and Harefield NHS Foundation Trust London UK; ^3^ Department of Histopathology Royal Brompton and Harefield NHS Foundation Trust London UK

**Keywords:** ADP, genetics, haemostasis, metabolomic, NSCLC, platelet activation, transcriptomics

## Abstract

Lung cancer is the leading cause of cancer‐related deaths in the world. The most prevalent subtype, accounting for 85% of cases, is non‐small‐cell lung cancer (NSCLC). Lung squamous cell carcinoma (LUSC) and lung adenocarcinoma (LUAD) are the most common subtypes. Despite recent advances in treatment, the low 5‐year survival rate of NSCLC patients (approximately 13%) reflects the lack of early diagnostic biomarkers and incomplete understanding of the underlying disease mechanisms. We hypothesized that integration of metabolomic, transcriptomic and genetic profiles of tumours and matched normal tissues could help to identify important factors and potential therapeutic targets that contribute to tumorigenesis. We integrated omics profiles in tumours and matched adjacent normal tissues of patients with LUSC (*N* = 20) and LUAD (*N* = 17) using multiple system biology approaches. We confirmed the presence of previously described metabolic pathways in NSCLC, particularly those mediating the Warburg effect. In addition, through our combined omics analyses we found that metabolites and genes that contribute to haemostasis, angiogenesis, platelet activation and cell proliferation were predominant in both subtypes of NSCLC. The important roles of adenosine diphosphate in promoting cancer metastasis through platelet activation and angiogenesis suggest this metabolite could be a potential therapeutic target.

**Abbreviations**
ADP: adenosine diphosphate
HMDB: Human Metabolome Database
LC/MS: liquid chromatography/mass spectrometry
LUAD: lung adenocarcinoma
LUSC: lung squamous cell carcinoma
NSCLC: non‐small‐cell lung cancer
WGCNA: weighted gene co‐expression network analysis

## 1 Introduction

Lung cancer is the leading cause of cancer‐related deaths in the world. The most prevalent subtype, accounting for 85% of cases of lung cancer, is non‐small‐cell lung cancer (NSCLC). Within NSCLC, lung squamous cell carcinoma (LUSC) and lung adenocarcinoma (LUAD) are the most prevalent subtypes, accounting for approximately 75% of cases. Despite major improvements in treatment options for NSCLC, such as checkpoint inhibitors (anti‐PD‐1/PD‐L1) and targeted therapies (tyrosine kinase inhibitors in relation to epidermal growth factor receptor (*EGFR*), anaplastic lymphoma receptor tyrosine kinase (*ALK*) and *ROS1* gene abnormalities), the 5‐year survival rate remains approximately 13% (Siegel *et al*., 2018). Poor survival is due to lack of early diagnostic biomarkers as well as the incomplete understanding of the underlying disease mechanisms.

Advances in genomics and genetics have enabled improved characterization of molecular subtypes of NSCLC. Lesions include *EGFR* mutation, and *ALK* and *ROS1* receptor fusion, which if present significantly improves targeted treatment outcomes of cancer patients. Besides these molecular mutations, abnormal cellular metabolism is also a hallmark of lung cancer (Hanahan and Weinberg, 2011). As with other types of cancer, the metabolic profile of NSCLC has been characterized by upregulation of key metabolic pathways such as glycolysis (Fahrmann *et al*., 2017), the TCA cycle (Fan *et al*., 2009), Krebs cycle (Sellers *et al*., 2015) and nucleotide metabolism (Moreno *et al*., 2018). Unfortunately, specific metabolic biomarkers of tumorigenesis and potential treatment targets are not well established.

Although metabolomic profiles in NSCLC have been characterized using plasma (Louis *et al*., 2017), serum (Kumar *et al*., 2017) and sweat samples (Calderon‐Santiago *et al*., 2015), the number of investigations into metabolomic profiles in the tumours themselves and their adjacent normal tissues is limited. Amongst these studies, Rocha *et al*. (2015) studied LUSC and LUAD tumours and found that in LUAD, phospholipid and protein metabolism were dominant, while glycolytic and glutaminolytic profiles were highly activated in LUSC tumours. More recently, Moreno *et al*. (2018) observed significant changes in glucose, glutathione, lipid and nucleotide metabolism in tumours in comparison with the normal tissues. So far, Fahrmann *et al*. (2017) are the only investigators to characterize and report potential interactions between metabolites and transcriptomes in NSCLC. It is important to note however that the transcriptomic data used by Fahrmann *et al*. were not derived from the same patients that underwent the metabolomic assay.

Comprehensive investigations into the underlying biochemical (metabolomic) and molecular (genomic and genetic) perturbations that accompany tumorigenesis have not been performed previously. We hypothesized that integration of the omics data would enable the identification of important key factors that contribute to tumorigenesis and factors that could be potential therapeutic targets for the treatment of NSCLC.

In this study, by combining metabolomic, genomic and genetics profiles of tumours and matched adjacent normal tissues of patients with LUSC (*N* = 20) and LUAD (*N* = 17), we have been able to investigate the relationships between metabolites, gene expression and tumour genetic variants.

## 2 Materials and methods

### 2.1 Study cohort

Paired lung biopsy samples (tumour and adjacent normal tissue) were obtained from 37 patients diagnosed with NSCLC. They are a subset of NSCLC patients who had tumour resection at the Royal Brompton Hospital between the years 2009 and 2011. All patients gave written informed consent for research on biobanked tissue under the ethical approval given to the RBH NIHR BRU Advanced Lung Disease Biobank (NRES reference 10/H0504/9) and Brompton and Harefield NHS Trust Diagnostic Tissue Bank (NRES reference 10/H0504/29). The study methodologies followed the standards set by the Declaration of Helsinki.

Tissue samples had been specifically collected and optimally stored (within 2 h after collection) for genomics, with tissue for transcriptomics stored in RNAlater (Qiagen, Crawley, UK) and tissue for genomic DNA and metabolomics snap‐frozen at the time of surgical resection and archived at −80 °C. Tumour histology and confirmation of histology subtype were through pathology review (A. Nicholson) of haematoxylin and eosin‐stained sections.

### 2.2 Metabolomics

Paired lung biopsy samples stored at −80 °C were sent to Metabolon for metabolomic profiling, as described previously (Moreno *et al*., 2018). Technical procedures included sample preparation, QA/QC, liquid chromatography/mass spectrometry (LC/MS, LC/MS^2^), GC/MS, accurate mass determination and MS/MS fragmentation, data extraction and quality assurance, compound identification and normalization.

#### 2.2.1 Sample accessioning

Each sample received was accessioned into the Metabolon Laboratory Information Management System (LIMS) and was assigned by the LIMS a unique identifier, which was associated with the original source identifier only. This identifier was used to track all sample handling, tasks, results, etc. The samples (and all derived aliquots) were bar‐coded and tracked by the LIMS. All portions of any sample were automatically assigned their own unique identifiers by the LIMS when a new task was created; the relationship of these samples was also tracked. All samples were maintained at −80 °C until processed.

#### 2.2.2 Sample preparation

The sample preparation process was carried out using the automated MicroLab STAR^®^ system from Hamilton Company (Birmingham, UK). Recovery standards were added prior to the first step in the extraction process for QC purposes. Sample preparation was conducted using a proprietary series of organic and aqueous extractions to remove the protein fraction while allowing maximum recovery of small molecules. The resulting extract was divided into two fractions: one for analysis by LC and one for analysis by GC. Samples were placed briefly on a TurboVap^®^ (Zymark, Hopkinton, MA, USA) to remove the organic solvent. Each sample was then frozen and dried under vacuum. Samples were then prepared for the appropriate instrument, either LC/MS or GC/MS.

#### 2.2.3 QA/QC

For QA/QC purposes, a number of additional samples are included with each day's analysis. Furthermore, a selection of QC compounds is added to every sample, including those under test. These compounds are carefully chosen so as not to interfere with the measurement of the endogenous compounds. Tables 1 and 2 describe the QC samples and compounds. These QC samples are primarily used to evaluate the process control for each study as well as aiding in the data curation.

**Table 1:** Description of Metabolon QC samples

Type	Description	Purpose
MTRX	Large pool of human plasma maintained by Metabolon that has been characterized extensively	Assure that all aspects of Metabolon process are operating within specifications
CMTRX	Pool created by taking a small aliquot from every customer sample	Assess the effect of a nonplasma matrix on the Metabolon process and distinguish biological variability from process variability
PRCS	Aliquot of ultrapure water	Process Blank used to assess the contribution to compound signals from the process
SOLV	Aliquot of solvents used in extraction	Solvent blank used to segregate contamination sources in the extraction

**Table 2:** Metabolon QC standards

Type	Description	Purpose
DS	Derivatization standard	Assess variability of derivatization for GC/MS samples
IS	Internal standard	Assess variability and performance of instrument
RS	Recovery standard	Assess variability and verify performance of extraction and instrumentation

#### 2.2.4 Liquid chromatography/Mass spectrometry (LC/MS, LC/MS^2^)

The LC/MS portion of the platform was based on a Waters ACQUITY UPLC and a Thermo‐Finnigan LTQ mass spectrometer, which consisted of an ESI source and linear ion‐trap (LIT) mass analyser. The sample extract was split into two aliquots, dried and then reconstituted in acidic or basic LC‐compatible solvents, each of which contained 11 or more injection standards at fixed concentrations. One aliquot was analysed using acidic positive ion optimized conditions and the other using basic negative ion optimized conditions in two independent injections using separate dedicated columns. Extracts reconstituted in acidic conditions were gradient‐eluted using water and methanol both containing 0.1% formic acid, while the basic extracts, which also used water/methanol, contained 6.5 mm ammonium bicarbonate. The MS analysis alternated between MS and data‐dependent MS^2^ scans using dynamic exclusion.

#### 2.2.5 Gas chromatography/Mass spectrometry (GC/MS)

The samples destined for GC/MS analysis were redried under vacuum desiccation for a minimum of 24 h prior to being derivatized under dried nitrogen using bistrimethyl‐silyl‐trifluoroacetamide. The GC column was 5% phenyl, and the temperature ramp is from 40 to 300 °C in a 16‐min period. Samples were analysed on a Thermo‐Finnigan Trace DSQ fast‐scanning single‐quadrupole mass spectrometer using electron impact ionization. The instrument was tuned and calibrated for mass resolution and mass accuracy on a daily basis. The information output from the raw data files was automatically extracted as discussed below.

#### 2.2.6 Accurate mass determination and MS/MS fragmentation (LC/MS, LC/MS/MS)

The LC/MS portion of the platform was based on a Waters ACQUITY UPLC and a Thermo‐Finnigan LTQ‐FT mass spectrometer, which had a LIT front end and a Fourier transform ion cyclotron resonance mass spectrometer back end. For ions with counts greater than 2 million, an accurate mass measurement could be performed. Accurate mass measurements could be made on the parent ion as well as fragments. The typical mass error was less than 5 p.p.m. Ions with less than two million counts require a greater amount of effort to characterize. Fragmentation spectra (MS/MS) were typically generated in a data‐dependent manner, but if necessary, targeted MS/MS could be employed, such as in the case of lower level signals.

#### 2.2.7 Bioinformatics

The informatics system consisted of four major components, the LIMS, the data extraction and peak‐identification software, data processing tools for QC and compound identification, and a collection of information interpretation and visualization tools for use by data analysts. The hardware and software foundations for these informatics components were the LAN backbone, and a database server running Oracle 10.2.0.1 Enterprise Edition.

#### 2.2.8 LIMS

The purpose of the Metabolon LIMS was to enable fully auditable laboratory automation through a secure, easy‐to‐use and highly specialized system. The scope of the Metabolon LIMS encompasses sample accessioning, sample preparation and instrumental analysis and reporting and advanced data analysis. All of the subsequent software systems are grounded in the LIMS data structures. It has been modified to leverage and interface with the in‐house information extraction and data visualization systems, as well as third‐party instrumentation and data analysis software.

#### 2.2.9 Data extraction and Quality assurance

The data extraction of the raw mass spec data files yielded information that could be loaded into a relational database and manipulated without resorting to BLOB manipulation. Once in the database, the information was examined, and appropriate QC limits were imposed. Peaks were identified using Metabolon's proprietary peak integration software, and component parts were stored in a separate and specifically designed complex data structure.

#### 2.2.10 Compound identification

Compounds were identified by comparison with library entries of purified standards or recurrent unknown entities. Identification of known chemical entities was based on comparison to metabolomic library entries of purified standards. As of this writing, more than 1000 commercially available purified standard compounds had been registered into LIMS for distribution to both the LC and GC platforms for determination of their analytical characteristics. The combination of chromatographic properties and mass spectra gave an indication of a match to the specific compound or an isobaric entity. Additional entities could be identified by virtue of their recurrent nature (both chromatographic and mass spectral). These compounds have the potential to be identified by future acquisition of a matching purified standard or by classical structural analysis.

#### 2.2.11 Curation

A variety of curation procedures were carried out to ensure that a high‐quality data set was made available for statistical analysis and data interpretation. The QC and curation processes were designed to ensure accurate and consistent identification of true chemical entities, and to remove those representing system artefacts, misassignments and background noise.

Metabolon data analysts use proprietary visualization and interpretation software to confirm the consistency of peak identification amongst the various samples. Library matches for each compound were checked for each sample and corrected if necessary.

#### 2.2.12 Normalization

**Figure 1:**
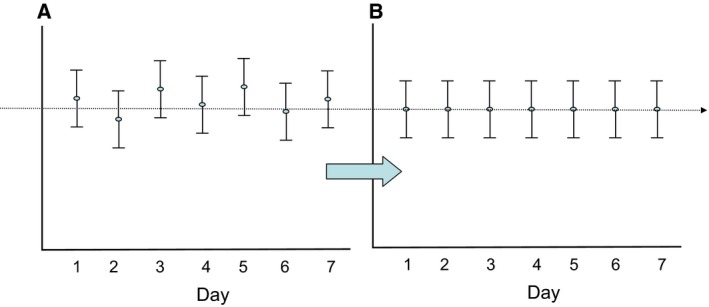
Visualization of metabolic data before (A) and after (B) normalization.

For studies spanning multiple days, a data normalization step was performed to correct variation resulting from instrument interday tuning differences. Essentially, each compound was corrected in run‐day blocks by registering the medians to equal one (1.00) and normalizing each data point proportionately (termed the ‘block correction’; Fig. 1). For studies that did not require more than 1 day of analysis, no normalization is necessary, other than for purposes of data visualization.

### 2.3 DNA and RNA extraction

Total genomic DNA was isolated from frozen tissue samples using a phenol–chloroform nonkit extraction method and PLG tubes (reagents from Sigma‐Aldrich, Dorset, UK, unless otherwise specified). Briefly to each tissue, resuspension buffer (0.075 m EDTA pH 8, 0.024 m NaCl, deionised distilled water) with 1% SDS (final concentration) was added, and then, homogenization was performed using the Qiagen TissueRuptor^®^ and disposable probes (Qiagen). Following an overnight 37 °C proteinase K digestion, phenol–chloroform extractions were performed using phase‐lock gel tubes. After ethanol precipitation of the DNA from the aqueous layer, DNA was pelleted by centrifugation and air‐dried at room temperature for 30 min prior to resuspension in 250 μL 10 mm Tris (Qiagen Elution Buffer). Yield and purity of genomic DNA obtained were assessed using a NanoDrop ND‐1000 spectrophotometer (NanoDrop; Thermo Scientific, Wilmington, DE, USA). DNA was stored at −20 °C until further use.

Total RNA was extracted from tissues (stored in RNAlater) using the Qiagen RNeasy Fibrous Tissue Midi Kit including the recommended homogenization step with the Qiagen TissueRuptor^®^ and disposable probes (Qiagen). Yield and purity of total RNA obtained were assessed using a NanoDrop ND‐1000 spectrophotometer (NanoDrop; Thermo Scientific) with RNA integrity determined by RNA integrity number using a Bioanalyzer 2100 (Agilent Technologies, Santa Clara, CA, USA). RNA was stored at −80 °C until further use.

### 2.4 Global gene expression: generation, quality control and preprocessing

Global gene expression data for each extracted RNA sample were generated using Affymetrix Human Gene 1.1ST arrays and the GeneTitan system following Affymetrix protocols (Affymetrix, Santa Clara, CA, USA). Briefly 200 ng of RNA amplified sense‐strand cDNA was generated using the Ambion^®^ WT Expression Kit (Life Technologies, Carlsbad, CA, USA). This was then fragmented and labelled using the Affymetrix GeneChip^®^ WT Terminal Labeling Kit (Affymetrix) before hybridization onto the arrays and subsequent scanning on the GeneTitan system. Poly‐A RNA controls were included as per Affymetrix's recommendations. Quality of the expression data generated was assessed through arrayQualityMetrics (3.30.0) and the relative log expression and normalized unscaled standard errors metrics calculated within the Bioconductor package Oligo (1.38.0). The data used for this integrative analysis were extracted from a subset of samples for which metabolomic, transcriptomic and genetic profiles were generated. Within this subset, no samples were identified by these collective metrics as being potentially problematic, and therefore, all 74 (37 pairs, tumour and normal) were retained in downstream analyses. Raw expression data for the samples were RMA‐treated using Oligo (1.38.0) and filtered. Specifically, transcript cluster (TC) intensity was required to exceed the data set median in 1 or more samples (Genefilter 1.56.0), and be designated within the Affymetrix annotation (NetAffx build 36) with a cross‐hybridization potential of 1 (unique), a nonmissing mRNA assignment and as part of the main design probe set category. Together these filters yielded 18 717 TC. Gene annotations were collated from the NetAffx build 36 and the Bioconductor package hugene11sttranscriptcluster.db (8.5.0) as assembled from public repositories. All the raw and normalized data were deposited to GEO (accession number: GSE134381).

### 2.5 Next‐generation sequencing of a targeted gene panel

A custom gene panel of 52 genes which was known to have mutation hotspots in cancer was designed based on published literature (Swanton and Govindan, 2016) (Berger *et al*., 2016; Campbell *et al*., 2016) and findings from prior in‐house whole‐exome sequencing of a set of 34 paired tumour and normal tissue NSCLC samples. The study was not powered to detect effects from rare mutations, but we sought for possible insights with common mutations such as those affecting *TP53*. The panel was designed using the Agilent software SureSelect DNA Advanced Design Wizard, based on the Human Genome February 2009 assembly (GRCh37/hg19). The panel focuses on the exonic regions of 52 genes that have been found recurrently mutated in NSCLC (Table S2). Sequencing libraries were prepared with the SureSelect QXT Target Enrichment System (Agilent) for the Illumina Multiplexed Sequencing platform (Illumina, San Diego, CA, USA) according to the manufacturer's instructions. Libraries were sequenced on an Illumina NextSeq 550 automated sequencer. fastqc software (version 0.11.5) (https://www.bioinformatics.babraham.ac.uk/projects/fastqc) provided assessment of the quality of the sequenced bases, and Phred scores were used to exclude low‐quality reads. Alignment against the Human Reference Genome December 2013 assembly (GRCh37/hg19) was performed using BWA mem (version 0.7.15), and Genome Analysis Tool Kit (GATK) (version 3.7) allowed local realignment around known insertions. Further, VarScan (version 2.4.2) was used for calling of somatic SNPs and indels by the analysis of matched tumour–normal samples, and gene annotation was obtained with Ensembl Variant Effect Predictor (version 92). Filtering of annotated variants was then carried out based on population‐level frequency (for known variants), gene‐level annotation and clinical impact. Variants were selected when they were observed at ≥ 1% frequency in tumour, when they had no associated frequencies in the dbSNP, 1000 Genomes, NHBLI ESP, Exome Aggregation Consortium and Genome Aggregation Database or when, if the variant was known already in population‐level databases, its observed incidence was < 0.001. At the functional impact level, only high and moderate variants were selected, or low‐impact variants when dbscSNVA predicted score was > 0.6. In addition, CADD score ≥ 15 was used and correlated with SIFT and PolyPhen prediction scores to predict potential protein‐damaging effects of missense variants. Suspected artefactual variants were manually checked using Integrated Genome Viewer to discard potential false positives. Specifically, variants were filtered out if the alternative allele did not show an approximate 50% breakdown in sense and antisense strands or if nearby nucleotides did not match the reference allele, to distinguish from sequencing noise. In addition, any SNV present ±3 bases from an indel was assumed to be a misalignment. Finally, all the variants (including high‐allele fraction variants) were checked manually on MutationTaster, COSMIC‐3D and cBioPortal to exclude polymorphic or known benign variants.

### 2.6 Statistical analysis

Paired Student's *t*‐tests were used to identify significant metabolites between the tumours and their matched normal tissue controls. Significant metabolites were defined to have Benjamini and Hochberg (BH)‐corrected *P*‐value ≤ 0.05 and fold change ≥ 1.5. Significance analysis of microarray was used to determine differentially expressed transcripts between the tumours and matched controls. Significant transcripts were defined to have false discovery rate (FDR) ≤ 0.05 and fold change ≥ 2.

### 2.7 Pathway and network analysis

Significant metabolites and transcripts were chosen for pathway and network analysis using MetaboAnalyst (Chong *et al*., 2018). Enrichment analysis was used to determine significant pathways from the predefined metabolite sets using metabolite set enrichment analysis (Xia and Wishart, 2010). For this analysis, a list of metabolites with Human Metabolome Database (HMDB) identifiers were used for over‐representation analysis (ORA) against the predefined library of 99 metabolite sets based on normal human metabolic pathways ( http://www.smpdb.ca). The top 50 most significantly enriched metabolic pathways were determined using ORA. The *P*‐value from ORA indicates the probability of seeing at least a particular number of metabolites from a certain metabolite set in a given compound list. MetaboAnalyst's network explorer analysis was used to explore relationships between the significant metabolites and transcripts. For this analysis, lists of significant genes and metabolites (including fold changes) from the same group were used to examine gene–metabolite interactions in search tool for interactions of chemicals. These associations are based on co‐mentions highlighted in PubMed Abstracts including reactions from similar chemical structures and similar molecular activities. Next, the metabolites and transcripts in this network were analysed using Reactome (Fabregat *et al*., 2018) pathway analysis. Cytoscape (Shannon *et al*., 2003) and InnateDB (Breuer *et al*., 2013) were used to visualize genes and gene–metabolite networks.

### 2.8 Weighted gene co‐expression network analysis (WGCNA)

Weighted gene co‐expression network analysis (Langfelder and Horvath, 2008) was used to investigate the relationship between transcripts and metabolites. Firstly, Pearson's correlation coefficients between all pairs of transcripts were calculated to form a correlation matrix of similarity. Next, a power (β) value of 6 was chosen to raise the co‐expression similarity. Co‐expressed gene modules, those with densely interconnected transcripts, were generated by unsupervised hierarchical clustering. The most highly interconnected modules with ≥ 35 genes were identified using dynamic branch cut method. Next, the correlation between module eigengene (value indicates module membership of each transcripts in the module) and key metabolites (traits) was calculated using the *GS* function. The most significant modules were further characterized by pathway and gene network analysis using InnateDB and Cytoscape.

## 3 Results

Thirty‐seven patients for whom pairs of tumour and adjacent normal tissues had undergone metabolomic, transcriptomic and genetic profiling were used for this analysis. Of these samples, 17 patients were histologically classified as LUSC and 20 were LUAD. Table 3 summarizes the demographic characteristics of the cohort. The male:female ratio was approximately 3 : 1 for both NSCLC subtypes. The median age at operation was 73 (59–85) years and 69.5 (61–89) years in LUSC and LUAD, respectively. Cancer stages (IA, IB, IIA, IIB and IIIA) were evenly represented in both groups. Finally, most patients were either current smokers or ex‐smokers.

**Table 3:** Social and pathological characteristics of the cohort. BMI: body mass index; *DDR2*: discoidin domain receptor tyrosine kinase 2

Characteristics	LUSC *N* = 17	LUAD *N* = 20
Gender (female, %)	5 (29.4%)	7 (35%)
Age at the time of operation	73 (59–85)	69.5 (61–89)
Cancer stage	IA: 6	IA: 5
	IB: 3	IB: 6
	IIA: 2	IIA: 3
	IIB: 3	IIB: 2
	IIIA: 3	IIIA: 4
DDR2 gene mutations	Not tested: 15 Wild‐type: 2	Not tested: 20
EGFR gene mutations	Not tested: 12 Wild‐type: 5	Not tested: 6 Wild‐type: 13 Mutant: 1
ALK fusion	Not tested: 12 No: 5	Not tested: 6 No: 13 Yes: 1
Smoking status	Current smoker: 6 Ex‐smoker: 11	Current smoker: 6 Ex‐smoker: 10 Never smoke: 3
BMI	26 (19–35)	26.5 (21–35)
Exposure to asbestos	Yes: 0 No: 6 Unknown: 11	Yes: 3 No: 4 Unknown: 13

### 3.1 Differences and similarities in metabolomic profiles between LUAD and LUSC

A total of 395 compounds were identified by metabolomic analysis. In comparison with normal tissue, 136 and 148 metabolites with known HMDB identifiers were statistically different in the tumours of LUSC and LUAD, respectively. Amongst these metabolites, 86 (43.4%) were significantly different in both LUSC and LUAD tumours when compared with their matched controls. Hierarchical clustering analysis showed a distinct profile in the tumour samples in relation to the controls (matched normal tissue) for both subtypes (Fig. S1).

**Figure 2:**
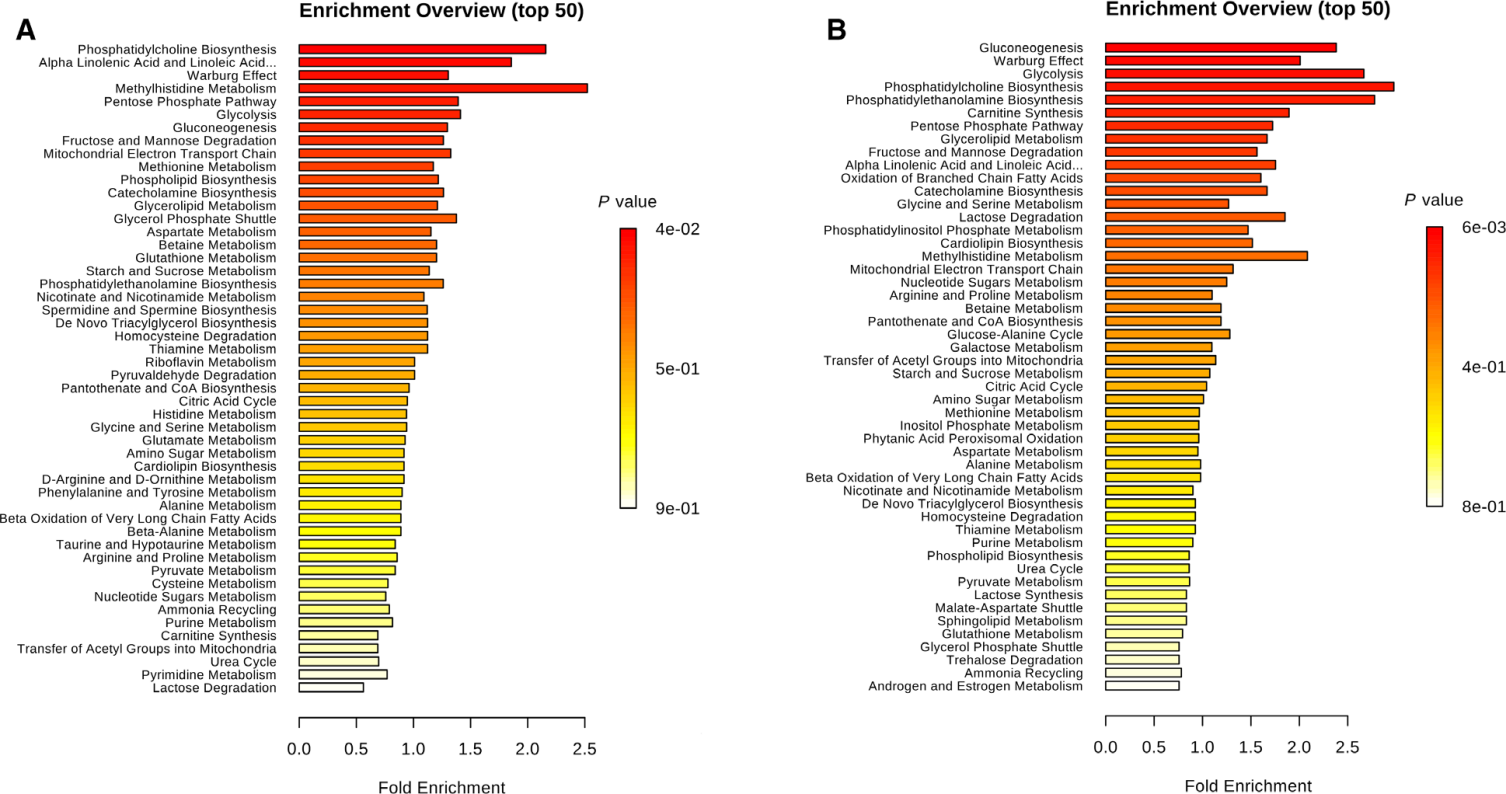
Metabolite enrichment analysis. Significant variation of metabolites in LUSC and LUAD tumours in comparison with matched normal tissues (adjusted *P*‐value ≤ 0.05, fold change ≥ 1.5). The top 50 most significant enriched pathways from 136 metabolites in LUSC (A) and 148 metabolites in LUAD (B) are shown. Similar enrichment profiles are seen in LUSC and LUAD tumours with the predominance of pathways affecting gluconeogenesis, the Warburg effect, glycolysis, pentose phosphate pathway and phosphatidylcholine biosynthesis.

Consistent with prior reports, our metabolite enrichment analysis revealed over‐representation of metabolites involved in gluconeogenesis, Warburg effect, glycolysis and nucleotide metabolism in both tumour subtypes (Fig. 2). The common metabolites that were involved in these pathways were d‐glucose, fructose 6‐phosphate, l‐lactic acid, phosphoenolpyruvic acid, 3‐phosphoglyceric acid, fructose 1,6‐bisphosphate, guanosine diphosphate, ADP, glucose 6‐phosphate and 2‐phospho‐d‐glyceric acid, l‐glutamic acid, l‐malic acid, succinic acid, phosphoenolpyruvic acid, fructose 1,6‐bisphosphate and 6‐phosphogluconic acid. Only two metabolites (1‐heptadecanoyl‐glycero‐3‐phosphocholine and 1‐C14:0‐lysophosphatidylcholine betaine) were significantly different between LUSC and LUAD tumours in direct comparison between these groups (Unpaired T test comparison of metabolites between LUSC and LUAD tumours).

### 3.2 Transcriptomic profile analysis

**Figure 3:**
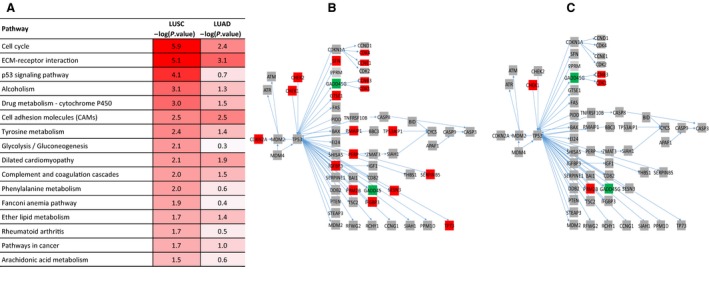
Reactome pathway analysis of significant transcripts. Significantly different transcript abundances in LUSC (*N* = 1979) and LUAD tumours (*N* = 931). The top 15 over‐represented pathways in LUSC (A) are shown. The level of significance is indicated by the intensity of red on the −log10 scale *P*‐value. Cell cycle and ECM‐receptor interaction signalling are the most significant in both LUSC and LUAD. However, p53 signalling is only significantly upregulated in LUSC tumours (B) and not in LUAD tumours (C). Genes that were found to be differentially expressed in the tumours in comparison with normal tissues are shown in red (more abundant) or green (less abundant). Genes that were found not to be significant are shown in grey.

In comparison with the matched normal tissues, there were 1979 and 931 transcripts differentially expressed in the LUSC and LUAD tumours, respectively. Reactome pathway analysis was performed to determine over‐represented pathways that were involved with these transcripts. We found that transcripts involved in cell cycle were the most over‐represented in both tumour subtypes (Fig. 3A). The p53 signalling pathway and its key elements such as *CDK2NA*,* CHEK2*,* CDK4*,* CCNE1*,* CCNB3*,* TP53AIP1*,* IGFBP3*,* SERPINB5*,* GTSE1* and *TP73* were highly upregulated in LUSC tumours when compared with their matched normal tissue controls (Fig. 3B) but not in LUAD tumours (Fig. 3C). We noticed that although glycolysis was significantly altered in metabolomic data in both LUSC and LUAD, the transcripts involved in this pathway were different.

### 3.3 Integration of transcriptomic and metabolomic data

**Figure 4:**
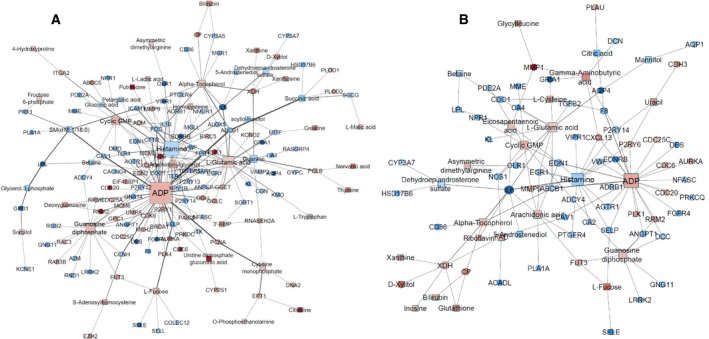
Metabolite–transcript interactions. MetaboAnalyst results for the interactions between the significant metabolites and transcripts in LUSC and LUAD. For LUSC, 153 metabolites and 1979 transcripts that were significant in the tumours form a network with 162 nodes (transcripts and metabolites) and 232 edges (A). Similarly, for LUAD tumours a network of 85 nodes and 116 edges is formed between the significant metabolites (*N* = 168) and transcripts (*N* = 931) (B). Network analysis by Cytoscape showed ADP, cyclin GMP, histamine, guanosine diphosphate and l‐glutamic acid as hub metabolites in both LUSC and LUAD. Genes (cycle) and metabolites (square) that were found to be more abundant in the tumours are shown in red with those less abundant shown in blue. The size of the nodes represents the degree of connectedness of genes or metabolites in the networks.

Next, we investigated the interaction between significant metabolites and transcripts using MetaboAnalyst. For LUSC, 153 metabolites and 1979 transcripts that were expressed at significantly different levels in the tumours formed a network with 162 nodes (transcripts and metabolites) and 232 edges (known interactions) (Fig. 4A). The expression pattern of the transcripts in this network is shown in Fig. S2. Similarly, a network of 85 nodes and 116 edges was formed between the significant metabolites (*N* = 168) and transcripts (*N* = 931) in LUAD tumours (Fig. 4B). Network analysis by Cytoscape showed ADP, cyclin GMP, histamine, guanosine diphosphate and l‐glutamic acid to be the hub metabolites for both the LUSC and LUAD networks.

Pathway analysis of the LUSC and LUAD networks by Reactome (summarized in Tables 4 and 5, respectively) revealed that haemostasis and platelet activation pathways were over‐represented in the tumours of both LUSC (*N* = 29 metabolites and transcripts) and LUAD (*N* = 26) subtypes. Metabolites significantly involved in these biological processes were ADP, arachidonic acid, Cyclic guanosine monophosphate (cGMP), FAD and guanine diphosphate, and significantly involved transcripts were *ANGPT1, PDE2A, A2M, VWF, GNG1, GNG14, GYPC, F8, CAV1, CFD, ITGAL, ITGA2 HGF, SELP, SELE, SELL OLR1, MGLL, OLR1, F10, CD36* and *TRPC6*. In addition, we found that transcripts involved in cell cycle regulation (*BRCA1*,* MSH2*,* PCNA*,* CD6*,* MCM3*,* MCM6* and *BIRC5*) were over‐represented and more abundant in LUSC tumours but not in LUAD tumours. The fold change of these genes was colour‐coded and is shown in Fig. 4. Survival analysis ( www.kmplot.com) using published data showed that expression level of PED2A and ANGPT1 was not associated with survival outcome in patients with LUSC. In contrast, in LUAD, high expression level of PDE2A was associated with poorer survival while high expression of ANGPT1 was associated with better survival outcome (Fig. 4).

**Table 4:** The top 24 most significant pathways of the metabolite–transcript interaction network for LUSC tumours

Pathway name	#Entities found	#Entities total	Entities ratio	Entities *P*‐value	Entities FDR
Signal amplification	7	42	0.003	4.88E‐06	0.004
ADP signalling through P2Y purinoceptor 1	6	29	0.002	7.03E‐06	0.004
Metabolism of nucleotides	15	254	0.018	1.15E‐05	0.004
Cyclin A/B1/B2‐associated events during G2/M transition	6	32	0.002	1.22E‐05	0.004
Nucleobase catabolism	11	139	0.010	1.29E‐05	0.004
Platelet activation, signalling and aggregation	16	293	0.021	1.52E‐05	0.004
Class A/1 (rhodopsin‐like receptors)	20	438	0.031	1.74E‐05	0.004
G alpha (i) signalling events	23	557	0.040	1.95E‐05	0.004
Haemostasis	29	812	0.058	2.30E‐05	0.004
Signal transduction	75	3158	0.226	2.75E‐05	0.004
Cell cycle, mitotic	23	570	0.041	2.79E‐05	0.004
DNA strand elongation	6	38	0.003	3.17E‐05	0.004
P2Y receptors	5	23	0.002	3.30E‐05	0.004
Activation of ATR in response to replication stress	6	39	0.003	3.66E‐05	0.004
GPCR ligand binding	24	629	0.045	4.47E‐05	0.004
Cell cycle	25	682	0.049	5.84E‐05	0.005
Nucleotide‐like (purinergic) receptors	5	28	0.002	8.30E‐05	0.007
Mitotic G1‐G1/S phases	11	173	0.012	9.08E‐05	0.008
G alpha (q) signalling events	14	274	0.020	1.05E‐04	0.008
MAPK1/MAPK3 signalling	14	280	0.020	1.31E‐04	0.010
Purine catabolism	7	74	0.005	1.70E‐04	0.012
GPCR downstream signalling	38	1344	0.096	1.81E‐04	0.012
FGFR1c and Klotho ligand binding and activation	3	7	0.001	1.89E‐04	0.012
Cell surface interactions at the vascular wall	13	256	0.018	1.96E‐04	0.012

**Table 5:** The top 24 most significant pathways of the metabolite–transcript interaction network for LUAD tumours

Pathway name	#Entities found	#Entities total	Entities ratio	Entities *P*‐value	Entities FDR
Haemostasis	26	812	0.058	1.95E‐09	2.85E‐06
Platelet activation, signalling and aggregation	15	293	0.021	2.19E‐08	1.60E‐05
Platelet degranulation	9	137	0.010	2.30E‐06	0.0011
Response to elevated platelet cytosolic Ca^2+^	9	144	0.010	3.43E‐06	0.0011
MAPK1/MAPK3 signalling	12	280	0.020	3.80E‐06	0.0011
Vasopressin regulates renal water homoeostasis via aquaporins	6	52	0.004	5.26E‐06	0.0013
RAF/MAP kinase cascade	11	273	0.020	1.73E‐05	0.0023
G alpha (q) signalling events	11	274	0.020	1.79E‐05	0.0023
MAPK family signalling cascades	12	331	0.024	2.00E‐05	0.0023
Integrin alphaIIb beta3 signalling	5	39	0.003	2.01E‐05	0.0023
Integrin signalling	5	39	0.003	2.01E‐05	0.0023
DCC‐mediated attractive signalling	4	19	0.001	2.09E‐05	0.0023
Nucleobase catabolism	8	139	0.010	2.19E‐05	0.0023
Aquaporin‐mediated transport	6	68	0.005	2.37E‐05	0.0023
FGFR1c and Klotho ligand binding and activation	3	7	0.001	2.97E‐05	0.0027
Purine catabolism	6	74	0.005	3.78E‐05	0.0032
Phosphorylation of Emi1	3	8	0.001	4.41E‐05	0.0036
Signalling by moderate kinase activity BRAF mutants	5	48	0.003	5.35E‐05	0.0038
Cell surface interactions at the vascular wall	10	256	0.018	5.49E‐05	0.0038
G alpha (i) signalling events	15	557	0.040	5.56E‐05	0.0038
Paradoxical activation of RAF signalling by kinase inactive BRAF	5	49	0.004	5.89E‐05	0.0039
Neurofascin interactions	3	9	0.001	6.24E‐05	0.0039
GPCR downstream signalling	25	1344	0.096	8.26E‐05	0.0048
Platelet aggregation (plug formation)	5	53	0.004	8.49E‐05	0.0048

### 3.4 Transcriptome–metabolite interactions revealed by WGCNA

To investigate correlations between gene expression and the level of metabolites, all the samples (*N* = 74) and their normalized expression data (*N* = 18 717 transcripts) were included in WGCNA. We identified 11 highly co‐expressed gene modules and calculated their correlation with key metabolites from the gene–metabolite interaction network (ADP, histamine, glutathione, cyclin GMP, guanosine diphosphate and l‐glutamic acid). The turquoise (2696 genes), blue (*n* = 1821) and green (*n* = 214) modules were significantly correlated with the level of ADP, glutamate, histamine, GDP‐fructose, 3′‐AMP and 5′‐AMP.

**Figure 5:**
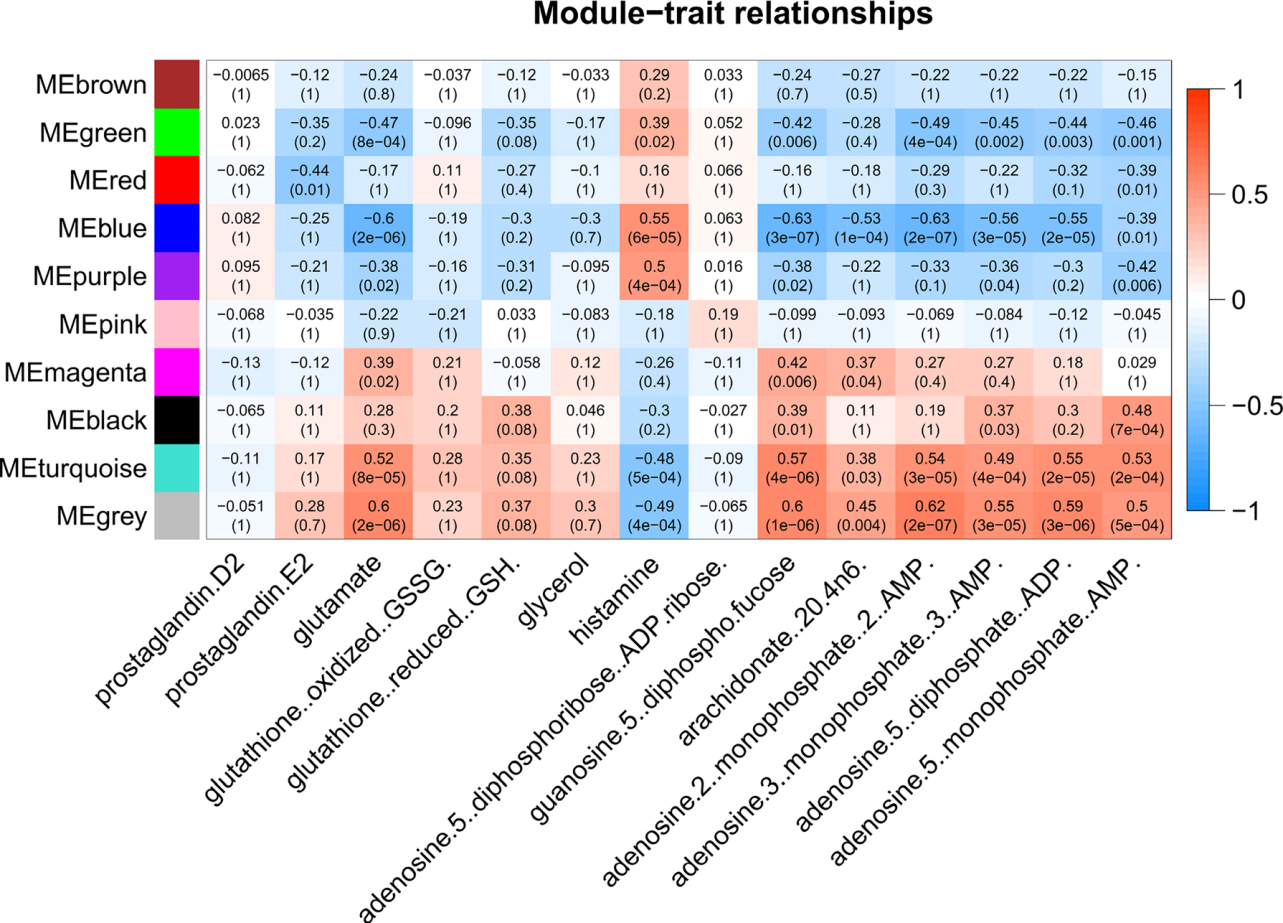
Transcriptome–metabolite interactions revealed by WGCNA. Correlation of WGCNA module eigengenes is shown with the key metabolites from the gene–metabolite interaction network. Gene modules turquoise (2696 genes), blue (*N* = 1821) and green (*N* = 214) were most significantly correlated with the level of the metabolites. Pearson's correlation coefficients are shown, with red representing positive and blue representing reversed correlation. The numbers in these boxes show actual correlation coefficient (top) and BH‐adjusted *P*‐value (bottom). ME, module eigengene.

**Figure 6:**
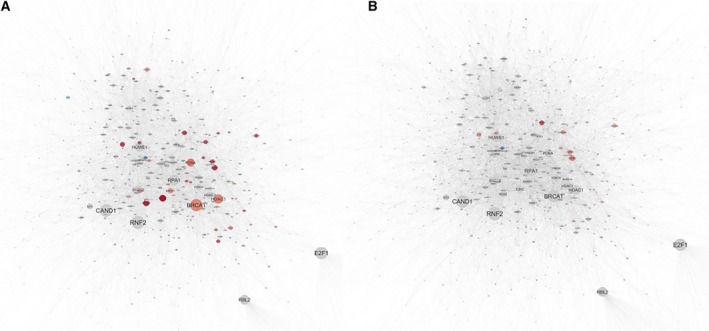
Turquoise gene module network and ontology analysis. Functional analysis by Reactome of the turquoise module showing over‐representation of genes in cell cycle, including G2/M checkpoints, mitotic, M phase and DNA repair. InnateDB gene network analysis visualized by Cytoscape shows hub genes that were significantly more abundant in LUSC (A) but not in LUAD tumours (B) relative to matched normal tissue. Each node represents a gene. The size of a node corresponds to number of interactions of a gene to other genes in the network. Genes that were significantly differentially expressed in the tumours in comparison with the normal matched tissues are shown in red (more abundant) or blue (less abundant). Genes that were not differentially expressed are shown in grey.

Functional analysis of the turquoise module by Reactome (Table S1) showed an over‐representation of cell cycle‐related genes, such as genes that encode proteins for the G2/M checkpoint, mitotic processes, M phase and DNA repair‐related genes. The transcripts in this gene module were expressed at a higher level in the tumour tissues of both LUAD and LUSC, with the greatest expression observed for LUSC tumours (Fig. 5). InnateDB gene network analysis (visualized by Cytoscape) revealed a highly connected gene network with 2088 nodes and 10 415 edges. Network analysis identified *BRAC1*,* HDAC1*,* PCNA*,* CDK1*,* EZH2* and *SOX2* as being the hub genes. When overlaid with statistical analysis, we found that these hub genes were only significantly more abundant in LUSC tumours (Fig. 6A) but not in LUAD tumours (Fig. 6B) relative to their matched normal tissue controls.

The green module was negatively correlated with the level of the key metabolites and included genes that were less abundant in tumours and dominant with genes involved in haemostasis and the platelet activation pathway.

### 3.5 Targeted sequencing of a custom cancer gene panel

**Figure 7:**
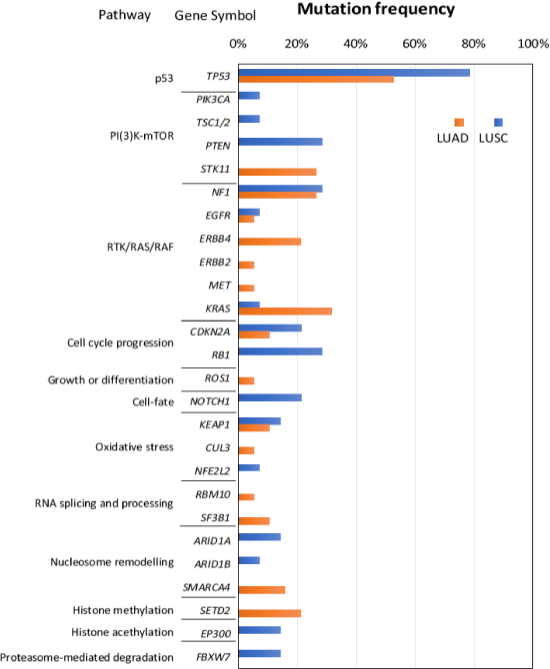
Amongst the 52 genes that were selected for targeted sequencing, 26 were shown to harbour mutations. *TP53* was found to be the most frequently mutated in both tumour subtypes (78% and 53% of LUSC and LUAD, respectively). Besides *TP53*, genes belonging to cell cycle progression such as *CDKN2A* and *RB1* were also mutated more frequently in LUSC tumours compared to LUAD tumours.

Amongst the 52 genes of the panel, alterations found in 26 were shown to harbour variants. The variants frequencies are summarized in Fig. 7. *TP53* (p53 signalling pathway) was the most frequently mutated in our cohort (78% and 53% in LUSC and LUAD, respectively). Genes of cell cycle progression such as *CDKN2A* and *RB1* were more frequently mutated in LUSC tumours. We did not see any effects of these common variants on individual metabolites.

## 4 Discussion

Energy metabolism is widely known to be aberrant in cancer (Hanahan and Weinberg, 2011), but detailed understanding of the metabolomics in NSCLC is limited. Differences in the metabolic profile between subtypes of NSCLC and its interactions with transcriptomics and genetic variant data in the same individuals have not previously been investigated. In this study, by integrating different types of these omics data, we were able to discover novel insights that were not detected when these data sets were analysed independently. Most importantly, metabolites and genes involved in haemostasis and platelet activation were prominent in both LUSC and LUAD tumours. The key roles of ADP, cGMP, GDP, histamine, glutathione and l‐glutamic acid in the gene–metabolite interaction network suggest they may have significant and important roles in disease mechanisms. In addition, cell cycle checkpoint genes (*PCNA, BRAC1, CD6, MCM3, MCM6* and *BIRC5*) and genes of the p53 signalling pathway (*CDK2NA, CHEK2, CDK4, CCNE1, CCNB3, TP53AIP1, IGFBP3, SERPINB5, GTSE1* and *TP73*) were strongly activated in LUSC but not in LUAD tumours. The higher mutation frequency of *TP53* in LUSC suggests interaction between these factors in this lung cancer subtype.

The limitation of our study was the relatively small sample size and lack of functional validations. However, the major strength of the study in comparison with the previous publications is the ability to integrate different omics data sets from tumours and the adjacent normal tissues from the same patients. These data sets allowed us to discover new insights into the interaction between the data sets and reveal new insights into the disease mechanism that was missed when these data sets were analysed separately.

An increased incidence of thromboembolic disease and haemostatic abnormalities is often observed in patients with cancer. There is considerable evidence that the haemostatic system is involved in the growth and spread of malignant disease. Platelets, beyond their role in haemostasis, may sustain tumorigenesis and metastasis via direct interaction with cancer and stromal cells and by the release of platelet products (Ballerini *et al*., 2018). Significant variation in the metabolites and transcripts identified by our study may therefore contribute to thromboembolic disease and metastasis of NSCLC.

Amongst the key metabolites, adenosine diphosphate (ADP), a pro‐angiogenic regulator and platelet agonist, is produced by cancer cells and by activated platelets. ADP is required for adhesion of platelets to cancer cells (Mitrugno *et al*., 2019), inducing platelet activation and aggregation through the purinergic P2Y1 and P2Y12 receptors. P2YR12 represents a potential target for an anticancer therapy due to its involvement in platelet‐cancer cell crosstalk, and P2YR12*‐*mediated platelet activation has been demonstrated to promote metastasis in mouse model of melanoma *(*Zhu *et al*., 2017 *)*. It has been previously speculated that manipulation of ADP and/or its receptor could limit cancer‐associated thrombosis (Murugappa and Kunapuli, 2006). Our data are consistent with the hypothesis that NSCLC tumours could promote their growth through platelet‐mediated angiogenesis.

An increase in haemostasis and platelet activation is supported by the downregulation of genes known to have inhibitory effect on haemostasis, such as *PDE2A* and *ANGPT1*. Platelets contain two cyclic adenosine monophosphate (cAMP) phosphodiesterases (PDEs) (PDE3A and PDE2A) that regulate the level of cAMP, a major platelet activation inhibitor. A recent study showed that the expression level (transcript) of *PDE2A* was significantly correlated with a microRNA called miR‐139, which is located within the intron of the *PDE2A* gene. In primary NSCLCs, decreased expression of miR‐139 was significantly associated with distant lymph node metastasis and histological invasiveness (lymphatic invasion and vascular invasion). The downregulation of *PDE2A* expression could therefore contribute to the increased risk of metastasis.

*ANGPT1* (encoding ANG1) is released upon platelet activation. In a mouse model, angiopoietin‐1/Tek signalling plays important roles in maintaining vascular integrity to limit metastasis (Michael *et al*., 2017). Previous studies have shown downregulation of *ANGPT1* in 80–95 % of oral squamous cell carcinomas (Jung *et al*., 2015). It is possible therefore that downregulation of *ANGPT1* may contribute to abnormal clotting in patients with NSCLC. Published data showed that LUAD patients with lower expression of ANGPT1 had poorer survival in comparison with those with high ANGPT1 expression.

Adenosine diphosphate may additionally inhibit endothelial cell proliferation by inducing cell cycle arrest in the S phase (Chen *et al*., 2018; Schafer *et al*., 2006). This inhibitory effect of ADP, acting through the *P2Y1* receptor, on cell proliferation has been observed in the mesothelioma cancer cell line ZL55. We also found that the key elements of p53 signalling pathway (such as *CDKN2A* together with other tumour suppressor genes such as *BRCA1*,* MSH2*,* PCNA* and *MCM3*) were not only silent in LUAD compared to LUSC tumours but were identical between LUAD tumours and their matched normal tissue. The effects of mutations in TP53 are complex, and gain‐of‐function effects are common (Schulz‐Heddergott and Moll, 2018). P53 affects multiple cellular processes, blocking cancer progression by inducing cellular growth arrest, promoting DNA repair and enabling programmed cell death. Therefore, we hypothesize that the increased production of ADP by cancer cells or activated platelets may provoke activation of the p53 signalling pathway. Although we observed higher mutation rates of TP53 in patients with LUSC, due to the scope of our study, we was unable to validate the effect of these mutations on the expression of p53 signalling pathway in the cohort. Finally, the contradictory biological functions (promoting angiogenesis and metastasis and inhibiting cell proliferation) and wide range of physiological effects of ADP in cancer progression suggest the importance of a balanced equilibrium between haemostasis, platelet activation and cell proliferation in the outcome of NSCLC and warrant further studies to provide more insights into these mechanisms.

We observed reduced levels of histamine, cGMP and GDP in tumours, which were negatively correlated with ADP. Histamine may be a crucial mediator in cancer development and progression. Its effects vary across cell types and depend on the balance between different receptor subtypes and its concentration within the tumour microenvironment (Massari *et al*., 2017; Stoyanov *et al*., 2012). Histamine levels in the plasma of NSCLC patients were found significantly decreased when compared with healthy controls (Della Rovere *et al*., 2006), consistent with our results. Although cigarette smoking is a possible confounder of the relationship between histamine and LUSC and LUAD (Della Rovere *et al*., 2006), only 12 out of 37 subjects were current smokers.

GTP metabolism is a main source of the RNA and DNA required for cancer cell proliferation. The transformation of GTP to GDP or *vice versa* is decided by either GTPase activating proteins or GTP exchange factors on their bounded GTPases and G proteins. A higher level of GDP but not GTP was accompanied by downregulation of GPCR‐related genes (*GNG11* and *GNA14*) in the tumours of both LUSC and LUAD. This perhaps indicates inactive forms of the GTPase and G proteins in the tumours. This finding suggests that NSCLC cells may have alternative strategies to promote proliferation and metastasis. cGMP is an important intracellular signal transduction molecule with both pro‐ and anticancer effects (Dhayade *et al*., 2016; Tinsley *et al*., 2009). For instance, cGMP has been shown to inhibit ADP‐induced platelet‐mediated angiogenic responses in the adenocarcinomic human alveolar epithelial cell line (A549) (He *et al*., 2017).

Because of our relatively small sample size, further studies are required to confirm the physiological impact of ADP, histamine, cGMP and GDP in NSCLC. Similarly, the relatively small sample size may have limited our power to relate variants associated with these metabolites.

## 5 Conclusions

In conclusion, our integrative analysis of metabolomic, transcriptomic and genomic data has discovered that metabolites and genes that contribute to angiogenesis and cell proliferation are predominant in both subtypes of NSCLC. The known important roles of ADP in promoting platelet activation and angiogenesis and in inhibiting cell proliferation suggest this metabolite and its receptors could be potential therapeutic targets for the treatment of NSCLC.

## Conflict of interest

The authors declare no conflict of interest.

## Author contributions

LH wrote the manuscript and performed data analysis; CDS performed targeted sequencing experiment; ES performed patient recruitment and sample preparation for metabolic assays; SWO performed transcriptomic data analysis; DMR supervised genetic and transcriptomic data analysis and contributed to revision of the manuscript; AN performed patient recruitment and revised the manuscript for important intellectual content; and WC and MM designed and conceptualize the study and wrote the manuscript.

## Supporting information


**Table S1.** Reactome Pathway Analysis of the Turquoise Module.
**Table S2.** Genes contained in the Agilent Gene Panel for targeted capture sequencing.
**Fig. S1.** Hierarchical clustering analysis of significant metabolites between tumour and matched normal control of LUSC (A) and LUAD (B).
**Fig. S2.** Hierarchical clustering analysis of transcripts that formed the metabolite–transcript network in Fig. 3A.
**Fig. S3.** Survival analysis ( www.kmplot.com) using published expression data of PED2A and ANGPT1 in lung cancer patients.

## Data Availability

Transcriptomic data are available for download from public repository (GEO accession number: GSE134381).
