# Supplementary material for: Metabolomic, transcriptomic and genetic integrative analysis reveals important roles of adenosine diphosphate in haemostasis and platelet activation in non‐small‐cell lung cancer
Source: Mol Oncol. 2019 Sep 30;13(11):2406–21. doi: 10.1002/1878-0261.12568 (PMC6822241; doi:10.1002/1878-0261.12568)
Supplement: Supplementary file 1 — Table S1. Reactome Pathway Analysis of the Turquoise Module. Table S2. Genes contained in the Agilent Gene Panel for targeted capture sequencing. Fig. S1. Hierarchical clustering analysis of significant metabolites between tumour and matched normal control of LUSC (A) and LUAD (B). Fig. S2. Hierarchical clustering analysis of transcripts that formed the metabolite–transcript network in Fig. 3A. Fig. S3. Survival analysis ( www.kmplot.com) using published expression data of PED2A and ANGPT1 in lung cancer patients. [file MOL2-13-2406-s001.pdf]

Supplementary materials

Metabolomics, transcriptomic and genetic - integrative analysis reveals important roles of adenosine diphosphate in haemostasis and platelet activation in non-small cell lung cancer

Supplementary Table 1: Reactome Pathway Analysis of the Turquoise Module

Pathway name	Entities				Reactions	
	found	ratio	p-value	FDR*	found	ratio
G2/M Checkpoints	88 / 154	0.011	1.11e-16	2.72e-14	24 / 24	0.002
Mitotic G1-G1/S phases	96 / 173	0.012	1.11e-16	2.72e-14	95 / 98	0.008
Cell Cycle, Mitotic	255 / 570	0.041	1.11e-16	2.72e-14	309 / 322	0.027
G1/S Transition	86 / 150	0.011	1.11e-16	2.72e-14	58 / 61	0.005
M Phase	158 / 390	0.028	1.11e-16	2.72e-14	62 / 66	0.006
Cell Cycle	294 / 682	0.049	1.11e-16	2.72e-14	379 / 411	0.035
Cell Cycle Checkpoints	151 / 279	0.02	1.11e-16	2.72e-14	40 / 57	0.005
DNA Replication	75 / 141	0.01	5.30e-14	1.05e-11	46 / 47	0.004
Separation of Sister Chromatids	92 / 194	0.014	5.53e-14	1.05e-11	8 / 8	6.79e-04
Mitotic Metaphase and Anaphase	97 / 211	0.015	6.93e-14	1.16e-11	12 / 12	0.001
Mitotic Anaphase	96 / 208	0.015	7.45e-14	1.16e-11	11 / 11	9.33e-04
Amplification of signal from unattached kinetochores via a MAD2 inhibitory signal	57 / 94	0.007	3.63e-13	4.80e-11	4 / 4	3.39e-04
Amplification of signal from the kinetochores	57 / 94	0.007	3.63e-13	4.80e-11	4 / 4	3.39e-04
DNA Replication Pre-Initiation	54 / 88	0.006	9.58e-13	1.17e-10	20 / 21	0.002
Synthesis of DNA	68 / 132	0.009	3.15e-12	3.37e-10	26 / 26	0.002
Mitotic Spindle Checkpoint	60 / 110	0.008	6.33e-12	6.39e-10	7 / 7	5.94e-04
S Phase	81 / 179	0.013	1.66e-11	1.57e-09	49 / 54	0.005
Chromosome Maintenance	57 / 105	0.008	2.51e-11	2.26e-09	27 / 27	0.002
Homology Directed Repair	63 / 130	0.009	2.13e-10	1.81e-08	48 / 50	0.004
Assembly of the pre-replicative complex	42 / 68	0.005	2.49e-10	2.02e-08	11 / 12	0.001
Resolution of Sister Chromatid Cohesion	64 / 134	0.01	2.72e-10	2.12e-08	8 / 8	6.79e-04
Nucleosome assembly	36 / 54	0.004	6.48e-10	4.60e-08	4 / 4	3.39e-04
Deposition of new CENPA-containing nucleosomes at the centromere	36 / 54	0.004	6.48e-10	4.60e-08	4 / 4	3.39e-04
Mitotic Prometaphase	84 / 207	0.015	1.18e-09	8.02e-08	15 / 15	0.001
G1/S-Specific Transcription	31 / 43	0.003	1.63e-09	1.03e-07	28 / 28	0.002

* False Discovery Rate

Supplementary Table 2: Genes contained in the Agilent Gene Panel for targeted capture sequencing. The gene panel consists of 12129 probes with a total size of 266.937 kbp.

AKT1	*EP300*	*MTAP*	*RIT1*
ALK	*ERBB2*	*MYC*	*ROBO1*
ARID1A	*ERBB4*	*MYCL*	*ROS1*
ARID1B	*FBBXW7*	*NF1*	*SETD2*
ARID2	*FGFR3*	*NFE2L2*	*SF3B1*
BRAF	*FHIT*	*NOTCH1*	*SMARCA4*
CCND1	*FOXP1*	*NRAS*	*SOX2*
CCND3	*HRAS*	*NTRK1*	*STK11*
CDK4	*KEAP1*	*PIK3CA*	*TERT*
CDKN2A	*KRAS*	*PTEN*	*TP53*
CREBBP	*MAP2K1*	*RB1*	*TSC1*
CUL3	*MDM2*	*RBM10*	*TSC2*
EGFR	*MET*	*RET*	*U2AF1*

Supplementary Figure 1

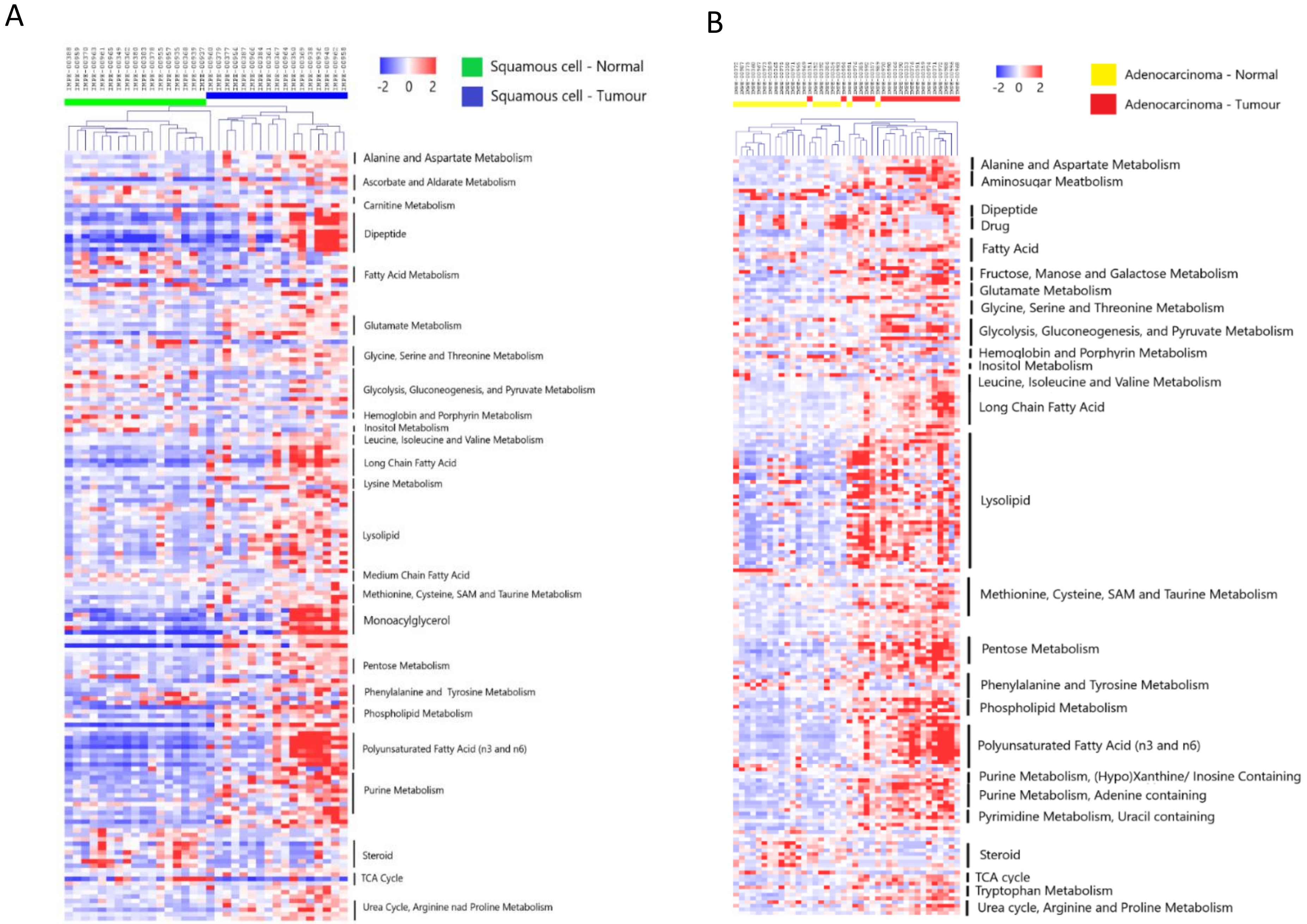

Supplementary Figure 2

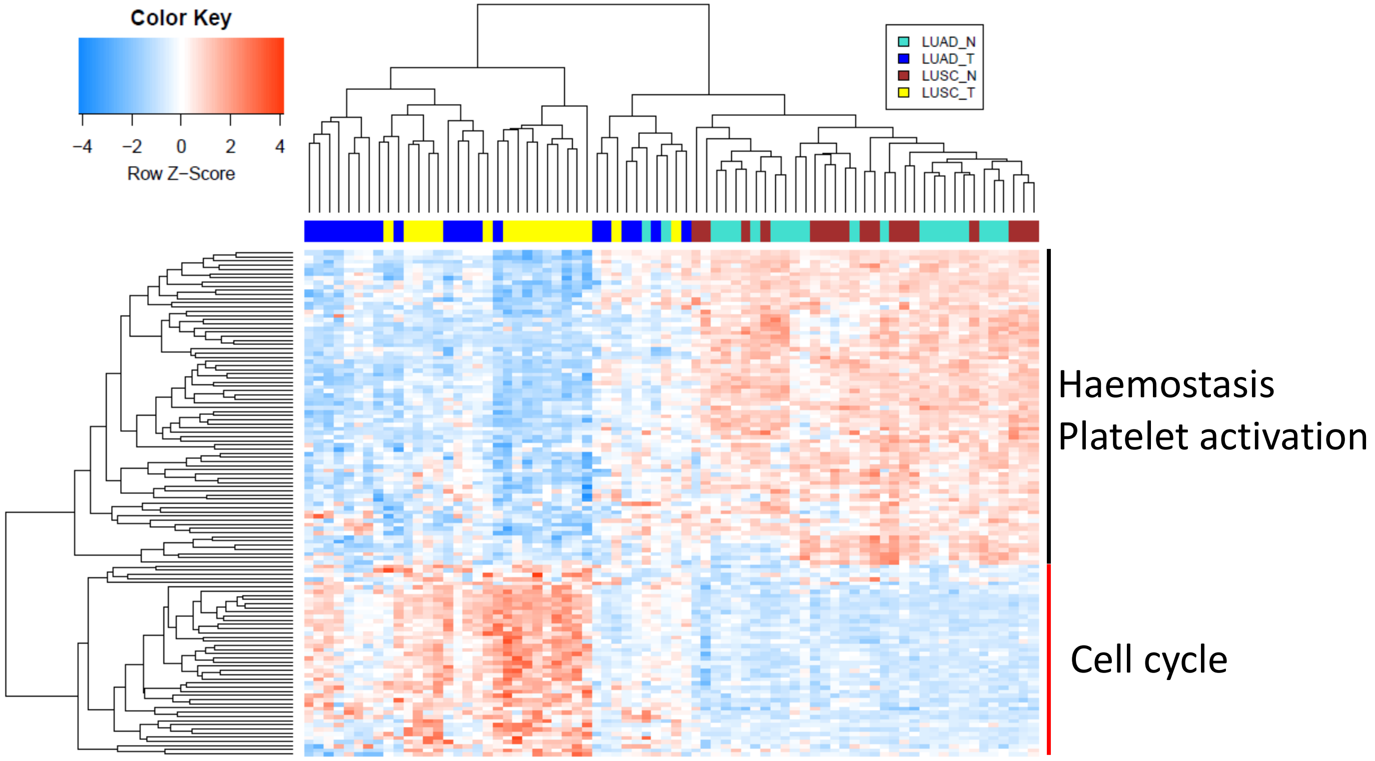

Supplementary Figure 3

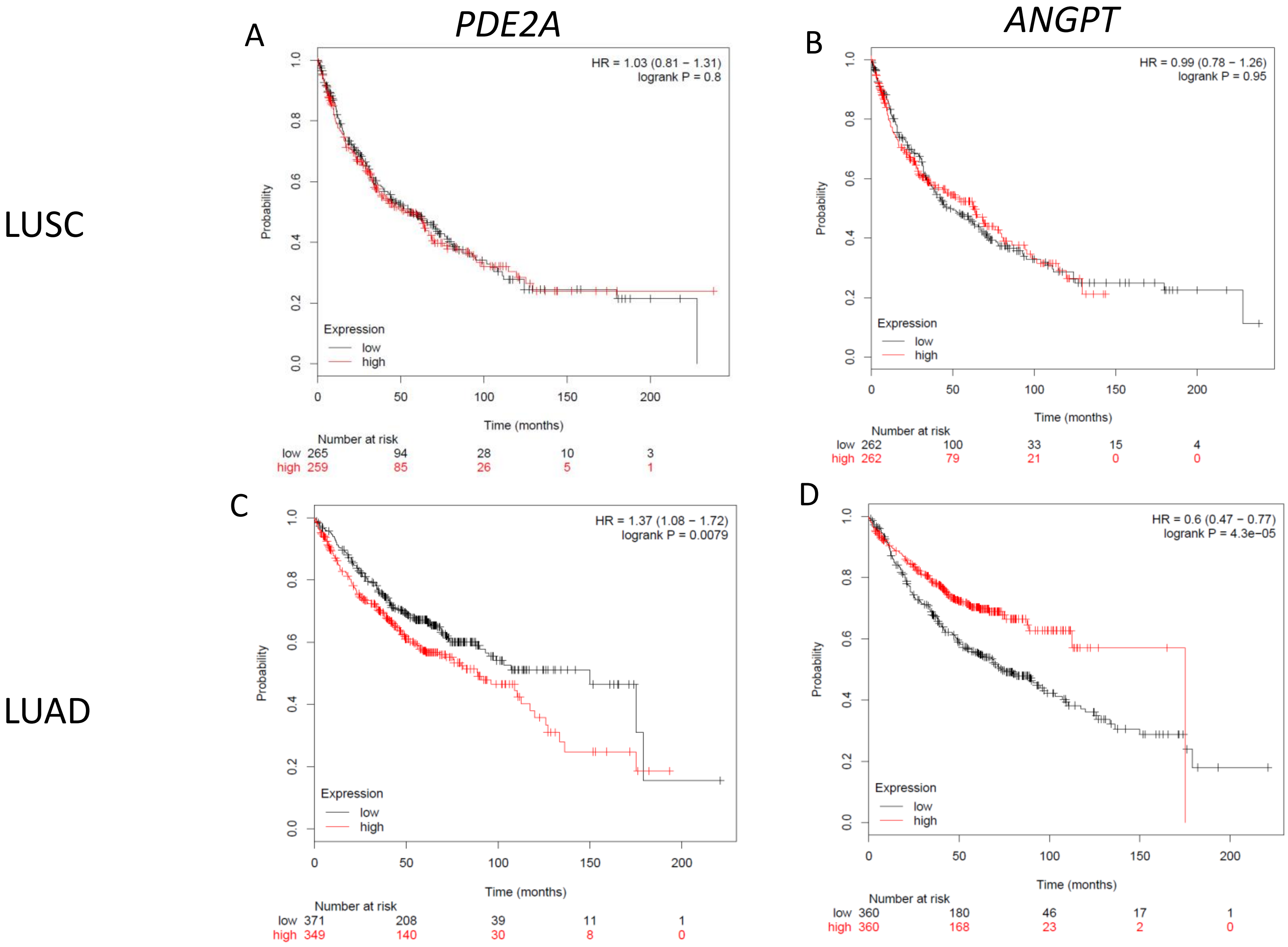

Supplementary Figure 1: Hierarchical clustering analysis of significant metabolites between tumour and matched normal control of LUSC (A) and LUAD (B). Metabolites with a higher level of expression in the tumours are shown in red or whilst those with a lower level of expression are indicated in blue.

Supplementary Figure 2: Hierarchical clustering analysis of transcripts that formed the metabolites – transcripts network in Figure 3A. Transcripts contributing to haemostasis and platelet activation were less abundant in both LUSC and LUAD tumours while those involving in cell cycle were more abundant. Transcripts with higher level in the tumours are shown in red or lower level in blue. LUAD-N: Normal tissue LUAD, LUAD-T: Tumour LUAD, LUSC-N: normal tissue LUSC, LUSC-T: Tumour LUSC

Supplementary Figure 3: Survival analysis (www.kmplot.com) using published expression data of *PED2A* and *ANGPT1* in lung cancer patients. The expression level of *PED2A* and *ANGPT1* was not associated with survival outcome in patients with LUSC (A and B). In contrast, in LUAD, high expression level of *PDE2A* was associated with poorer survival (C) while high expression of *ANGPT1* was associated with better survival outcome (D).
